# Design of novel orthotic insoles based on partition infilling of TPMS structures

**DOI:** 10.3389/fbioe.2026.1820362

**Published:** 2026-05-01

**Authors:** Yongtao Lyu, Lingqi Meng, Hao Wang, Zihao Wang, Lingyi Cong, Sergei Bosiakov, Yuanfei Ren

**Affiliations:** 1 The first Department of Hand and Foot Surgery, Central Hospital of Dalian University of Technology, Dalian, China; 2 DUT-BSU Joint Institute, Dalian University of Technology, Dalian, China; 3 Department of Engineering Mechanics, Dalian University of Technology, Dalian, China; 4 Faculty of Mechanics and Mathematics, Belarusian State University, Minsk, Belarus

**Keywords:** diamond, gyroid, primitive, orthotic insoles, partition infilling

## Abstract

**Introduction:**

Excessive plantar pressure has been recognized as a key risk factor for diabetic foot ulceration.

**Methods:**

To address this problem, a partition-infilled functional insole based on triply periodic minimal surface (TPMS) lattice structures was proposed and systematically investigated. First, the mechanical responses of three representative TPMS structures, namely Gyroid, Diamond and Primitive, were characterized by compression experiments and finite element (FE) analysis. Subsequently, a partition infilling strategy was designed according to distribution of the plantar pressure. Under additive manufacturing constraint of minimal thickness of 0.2 mm, different TPMS lattices were assigned to specific plantar regions.

**Results:**

Gait experiments and FE results demonstrated that, compared to a uniformly Gyroid-infilled insole and a single-lattice gradient-infilled insole, the partition-infilled insole achieved significant reductions in both peak and mean pressures. A Primitive lattice in the heel region exhibited superior pressure-relief performance, whereas Gyroid and Diamond lattices in the forefoot and midfoot balanced cushioning with overall structural stability. Owing to the mathematical definitions of different TPMS structures, smooth topological transitions between lattices were enabled, further improving comfort and manufacture ability. An integrated design-manufacturing framework for personalized diabetic insoles was established.

**Discussion:**

The potential of TPMS partition infilling for redistributing plantar loads and reducing ulceration risk was verified, providing theoretical and experimental support for subsequent clinical applications and large-scale additive manufacturing.

## 1 Introduction

Diabetes mellitus (DM) (Almalki and Khan, 2025) has been regarded as one of the most concerning global diseases due to the heavy economic burden imposed on healthcare systems (Aghaei Zarch et al., 2020; Hossain et al., 2024; Kirigia et al., 2009). By 2025, the global number of DM patients has reached 589 million (Xiao et al., 2025) and the number continues to rise annually. Diabetic foot (DF) has been the main cause of disability and mortality among DM patients, with approximately 15% of patients experiencing DF during the course of the disease (Rubio et al., 2020; Wang et al., 2020). Diabetic foot ulcer has been its most common manifestation, affecting the skin and deep tissues distal to the ankle and often accompanied by infection or various degrees of lower-limb arterial occlusion (Parveen et al., 2025; Wang et al., 2022). Ulcers and other deep-tissue lesions have been caused by high tissue pressure. Orthotic insoles (Bellomo et al., 2022; Ren et al., 2024) have been used to prevent and mitigate ulcers and deep-tissue lesions, thereby reducing the risk of infection spreading throughout the entire foot.

Recent progress in orthotic insoles has been driven by additive manufacturing. From a cost perspective, three-dimensional (3D) printing has enabled more economical production of customized insoles, minimized material waste, and made small-batch or individualized fabrication cost-effective (Kumar Panda et al., 2023; Tao et al., 2025). The demand for excessive tooling and molds, which are often expensive in traditional processes, has been reduced. The production process for 3D printing has gradually been developed (Calzado et al., 2019; MacDonald and Wicker, 2016). Once the structural design has been completed, only minimal manual adjustment has been required. This automation has greatly reduced the time needed for custom insoles (Mandolini et al., 2017), thereby improving operational efficiency. Rapid prototyping and fast iteration have shortened the total time for design modification and production, enabling faster delivery of personalized insoles.

Lattice structures have been widely used as infill structures for improving the mechanical performances of orthotic insoles (Leung et al., 2022; Sun et al., 2024; Lu et al., 2025). The cell size and wall thickness of lattice parameters, have been adjusted to reach different stiffnesses. Recent studies have further demonstrated the biomechanical advantages of optimized 3D-printed lattices. For instance, numerical homogenization and optimization algorithms have been utilized to tailor polymeric Gyroid structures across distinct foot regions, which effectively minimized the standard deviation of plantar pressures and significantly lowered peak stresses (Cracknell et al., 2025). Furthermore, solid-liquid composites incorporated with fluid-filled lattices have been proven to enable dynamic pressure redistribution and superior energy dissipation compared to traditional non-fluid-filled designs (Cracknell et al., 2024). According to plantar loading characteristics (Ha et al., 2023), the system recorded the effective elastic modulus of the Gyroid structure. It made dynamic adjustments to the lattice size, thickness and center offset across foot regions. Currently, the research on using different TPMS structures to infill various insole partitions remains rare. The various TPMS structures, including Primitive, Gyroid, and Diamond, have exhibited distinct initial elastic modulus and different mechanical advantages under the same geometric parameters (Qiu et al., 2024). Therefore, infilling various TPMS structures in different insole regions may be an excellent method to achieve functional requirements.

In this study, various TPMS structures were infilled into specific areas of the insole based on the load-bearing characteristics and functional requirements of those regions. Because TPMS structures govern by trigonometric functions (Lazar et al., 2023), transitions between different TPMS structures enable by a Sigmond function (Vijayavenkataraman et al., 2018). To address the problem of excessive concentration of plantar pressure, appropriate adjustment of the internal infilled structures in the entire insole was performed. The entire insole was divided into four partitions according the distribution of plantar pressure. Three TPMS structures (i.e., Gyroid, Diamond, and Primitive) were infilled into the different partitions of the insole due to their different mechanical properties. This strategy can effectively reduce the peak pressure compared the insoles without partitioning. Therefore, the study on partition-infilling with different TPMS structures may mitigate the deep tissue lesions, and provide insight for the prevention and treatment of DF.

## 2 Materials and methods

**FIGURE 1:**
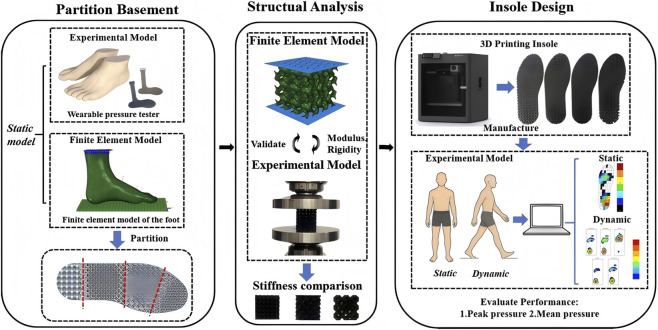
Schematic diagram of the research methodology.

In this work, the investigation on partition-infilling of the insole with different TPMS structures were conducted. To determine the optimal structure for each insole location, the distributions of plantar pressure were obtained through the results of the experiments and simulations. The quasi-static compression of different TPMS lattices was also studied, and the effective elastic modulus was of interest. The plantar surface was partitioned into toe, forefoot, midfoot, and heel (Queen et al., 2009). In heel region, lattices with the lowest stiffness and largest deformation under the same load were infilled, enlarging the foot–insole contact area to reduce peak pressure. In forefoot region, lattices with relatively low stiffness were infilled to lower peak and mean pressures while ensuring adequate support. In toe and midfoot regions, lattices with the highest stiffness were infilled to maintain the stability of the entire insole and to stabilize the foot during daily activities. An illustration of the methodology is shown in Figure 1.

### 2.1 FE modeling of the foot and determination of plantar pressure

#### 2.1.1 Geometry and meshing

To create the FE model of the foot, computed tomography (Siemens SOMATOM Definition 64-slice) image data of a normal foot from a volunteer with a weight of 72.0 kg were used. The image data was processed in Mimics (Version 21.0, Materialise, Belgium) to generate the 3D bone model of the foot, which was then smoothed in Geomagic Studio (Version 2014, 3D Systems Inc., Rock Hill, SC, United States). The tendons, ligaments and cartilage were ignored and other soft tissue of foot model was simplified as bulk material. Previous studies have proven the feasibility of foot finite element (FE) models constructed using such simplified methods (Beillas et al., 2004; Cheung et al., 2005). The foot model was meshed using Altair HyperMesh (Version 2021, Altair Engineering Inc., Troy, MI, United States). Both bone and soft tissue were discretized using C3D4 elements. A mesh convergence study was performed to ensure the mesh convergence was achieved.

#### 2.1.2 FE simulation of the interaction between foot and ground

**FIGURE 2:**
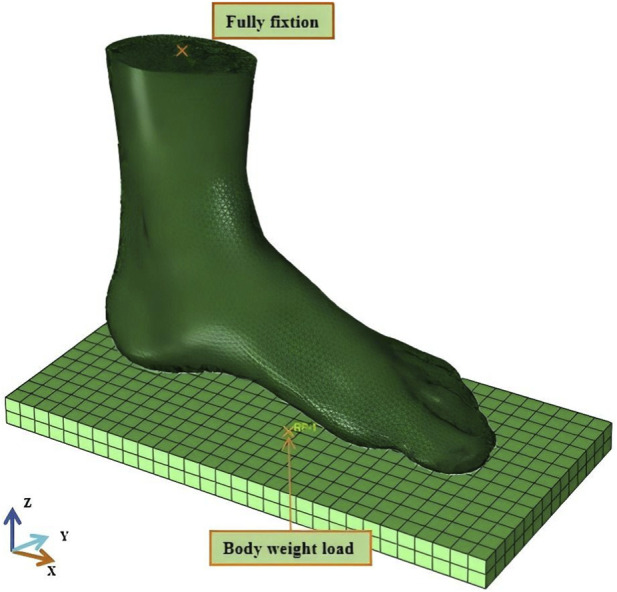
Finite element model of the foot and boundary conditions.

Based on the FE foot model, the interaction between the foot and ground was simulated. Material properties were defined, as shown in Table 1. Bones were modeled as linear elastic material. Soft tissue was modeled as a second-order polynomial hyperelastic material. The ground was regarded as rigid body. The interaction between the ground and the foot was set to frictional contact with a coefficient of 0.4. The scenario of natural standing posture was simulated. Therefore, for a 72.0 kg subject, half body weight was applied through the ground and the average load on one foot was 353.0 N. The upper end of the foot model was fully fixed, as shown in Figure 2. FE analysis was conducted using Abaqus (Version 2022, Dassault SIMULIA, Providence, RI, United States).

**TABLE 1:** Material models of each component (Kathirgamanathan et al., 2019).

Component	Constitutive model	Material properties
Bone	Linear elastic material	*E* = 7300 MPa *ν* = 0.3
Soft tissue	Second-order polynomial hyperelastic material	*C*_10_ = 0.08556, *C*_01_ = −0.05841, *C*_20_ = 0.03900, *C*_11_ = −0.02319, *C*_02_ = 0.00851, *D*_1_ = 3.165,273, *D*_2_ = 0.0000
Ground	rigid bodies	∞

#### 2.1.3 Determination of plantar pressure for partitioning

The plantar pressure was determined and used to design the insole partition strategy. A combined approach of experimental testing and numerical analysis was adopted. The distributions of plantar pressure were acquired using a Pedar-X pressure measurement system (Novel GmbH, Munich, Germany) during static standing tests. Walking tests were then conducted at 50.0 Hz to capture the full gait cycle. Sensor calibration was performed using the subject’s body weight as a parameter so that accurate data were ensured. The repeated tests were conducted to minimize the error in the plantar pressure.

### 2.2 Additive manufacturing and quasi-static compression of TPMS lattices

#### 2.2.1 Additive manufacturing of TPMS lattices

Fused deposition modeling (FDM) with TPU 95A HF material was adopted to manufacture the TPMS structures. Three TPMS structures (i.e., Gyroid, Diamond, and Primitive) were defined by trigonometric equations, as below:

$${\varphi \left(r\right)}_{G}=\sin\left(X\right)\cos\left(Y\right)+\sin\left(Z\right)\cos\left(X\right)+\sin\left(Y\right)\cos\left(Z\right)=C$$ (1)

$${\varphi \left(r\right)}_{D}=\cos\left(X\right)\cos\left(Y\right)\cos\left(Z\right)-\sin\left(X\right)\sin\left(Y\right)\sin\left(Z\right)=C$$ (2)

$${\varphi \left(r\right)}_{P}=\cos\left(X\right)+\cos\left(Y\right)+\cos\left(Z\right)=C$$ (3)

where *C* is a scaling factor, used to adjust the cell size and zero center offset was used. The TPMS lattices of 30.0 mm × 30.0 mm × 30.0 mm with a unit cell of 10.0 mm × 10.0 mm × 10.0 mm and a wall thickness of 0.6 mm were designed. The three TPMS structures can be expressed by Equations 1–3. The geometries of TPMS lattices were created using nTopology (Version 4.10.2, nTopology Inc., New York, NY, United States), and then printed in the vertical orientation using Bambu Lab P1S (Shenzhen, China).

#### 2.2.2 Quasi-static compression of TPMS structures

**FIGURE 3:**
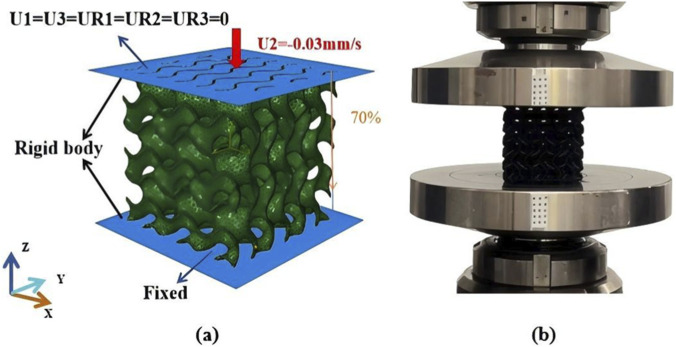
**(a)** Quasi-static compressive simulation of TPMS lattices; **(b)** Quasi-static compressive experimental tests of TPMS lattices.

Quasi-static compression of the TPMS structures were conducted using Abaqus to calculate stiffness of the structure. All structures were compressed to a strain of 70.0%, as shown in Figure 3a. General contact was used to capture self-contact. Both top and bottom plates were simulated as rigid bodies. The displacement was imposed on the top plate, and the bottom plate was completely fixed. Additionally, the friction coefficient for the tangential behavior in the compression direction was set to 0.2, and the normal behavior was that of “hard” contact.

#### 2.2.3 Quasi-static compression tests of TPMS structures

To evaluate mechanical properties of three TPMS structures, quasi-static compression tests were performed on a universal material testing machine with a measuring range of 10.0 kN. A constant loading rate of 1.0 mm/min was used, and specimens were compressed to a strain of 70.0%, as shown in Figure 3b. The force–displacement curves of three TPMS structures were recorded and the stress–strain relations were calculated from the force–displacement curves. Three specimens were prepared for each TPMS structure.

### 2.3 Design and evaluation of TPMS-infilled insoles

#### 2.3.1 Design of TPMS infilled insoles and fabrication

**FIGURE 4:**
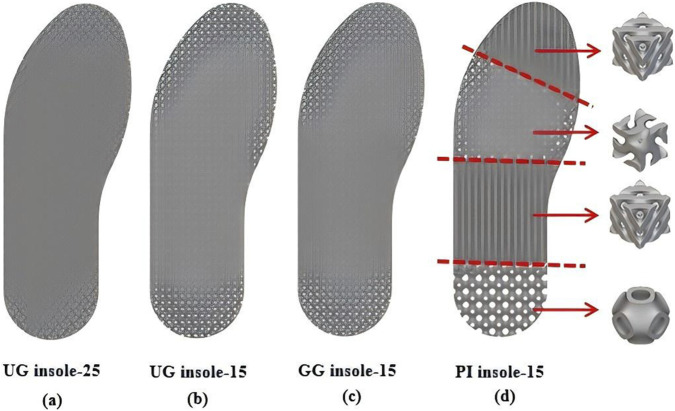
**(a)** Dimensions and geometries of the four 3D printed insoles: **(a)** UG insole-25; **(b)** UG insole-15; **(c)** GG insole-15; **(d)** PI insole-15.

The partition-infilled (PI) insole was designed using three TPMS structures. Different TPMS structures were infilled to various partitions according to the distributions of plantar pressure and the mechanical properties of the TPMS structures. To enable a smooth connection between two adjacent TPMS structures at transition boundaries, a Sigmoid function was used:

$$\alpha \left(x,y,z\right)=\frac{1}{1+e^{-kG\left(x,y,z\right)}}$$ (4)

where *α*(*x, y, z*) is the regional transition weighting function, *k* is a parameter controlling the width of the transition area, and *G* (*x, y, z*) defines the signed distance field (SDF) to the predefined partition boundary (Feng et al., 2022). Specifically, *G* (*x, y, z*) = 0 represents the exact geometric interface between two adjacent partitions, while its absolute value indicates the shortest distance from any spatial point to this boundary. A gradient-control parameter was established to investigate the connection at the interface. Therefore, smooth connection between two adjacent TPMS structures was achieved. The transition function can be expressed by Equation 4. The PI insole with a volume fraction of 15.0% (PI insole-15) was designed. Moreover, for comparison purpose, uniform and graded Gyroid structures (UG and GG) were also respectively infilled to the insoles, which also have a volume fraction of 15.0% (UG insole-15 and GG insole-15). The wall thicknesses of unit cells of graded Gyroid insole transitioned from thick in low-pressure regions to thin in high-pressure regions. Additionally, the UG infilled insole with a volume fraction of 25% (UG insole-25) was also generated to demonstrate the importance of the volume fraction. The Gyroid structure was chosen because many studies have demonstrated its suitability for orthotic insoles (Jonnala et al., 2023; Rico-Baeza et al., 2023). Four types of orthotic insole were generated in nTopology, as shown in Figure 4. The length and the thickness of all insoles were 255.0 mm and 10.0 mm, respectively. The dimension of all unit cells infilled in this study was 10.0 mm × 10.0 mm × 10.0 mm. Three Gyroid-based insoles served as references to demonstrate the superiority of the partition-infilled design. These insoles were fabricated using the same 3D printing technique as described in Section 2.2.1.

#### 2.3.2 Evaluation of the performance of orthotic insoles

**FIGURE 5:**
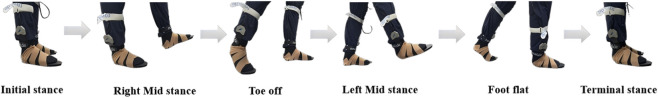
Plantar pressure at different gait phases.

Both the static and dynamic behaviors of the orthotic insoles were evaluated. A Pedar-X pressure measurement system was used for static standing and walking scenarios to obtain the distributions of plantar pressure. Walking tests were performed at 50.0 Hz to capture the full gait cycle precisely, as shown in Figure 5. Repeated tests were conducted to minimize errors of gait. Natural gait patterns were captured, and test data were used to identify each phase of the gait cycle according to known contact patterns from prior studies.

## 3 Results

### 3.1 Quasi-static compression of TPMS structures

**FIGURE 6:**
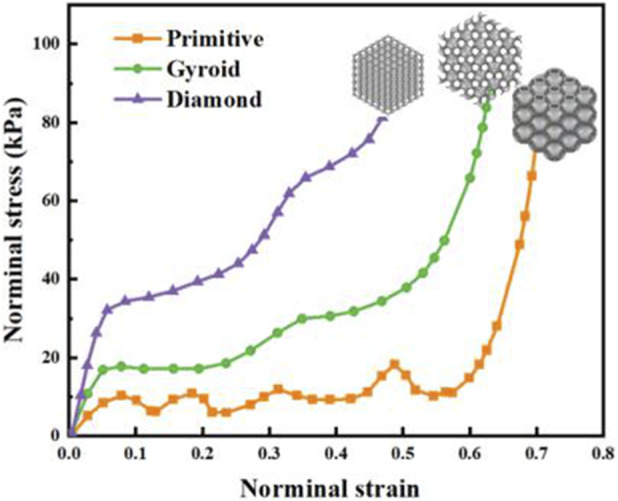
Engineering stress–strain curves from quasi-static compression simulations of Primitive, Gyroid, and Diamond lattices.

**FIGURE 7:**
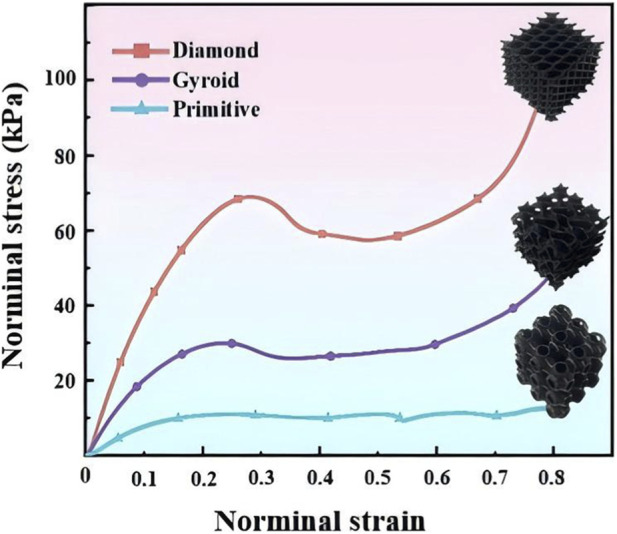
Normal stress–strain curves from quasi-static compression tests.

The quasi-static compressive FE simulations and experimental tests of three TPMS structures were conducted. Engineering stress–strain curves from FE simulations and experimental tests for Primitive, Gyroid, and Diamond with the same volume fraction were obtained, as shown in Figures 6, 7. The trends of FE results for the three TPMS structures were identical to those of experimental results. Mechanical responsive curves of TPMS structures were analyzed and divided into linear-elastic, plateau, and densification stages. Notably, under small strains, the initial elastic modulus was the highest for the Diamond structure, followed by the Gyroid structure, and the lowest for the primitive structure. Hence, the Primitive lattice exhibited the lowest stiffness and the largest deformation under the same compressive loading. The Gyroid lattice exhibited intermediate modulus. The Diamond lattice exhibited the highest stiffness and the smallest strain under the same compressive loading, implying that its high stiffness could be exploited to ensure entire insole support.

The TPMS structures were infilled into different partitions according to their mechanical properties. The Gyroid lattice with relatively low stiffness was infilled into the forefoot to expand the contact area while maintaining support.

### 3.2 Analysis of plantar pressure for insole partitioning

**FIGURE 8:**
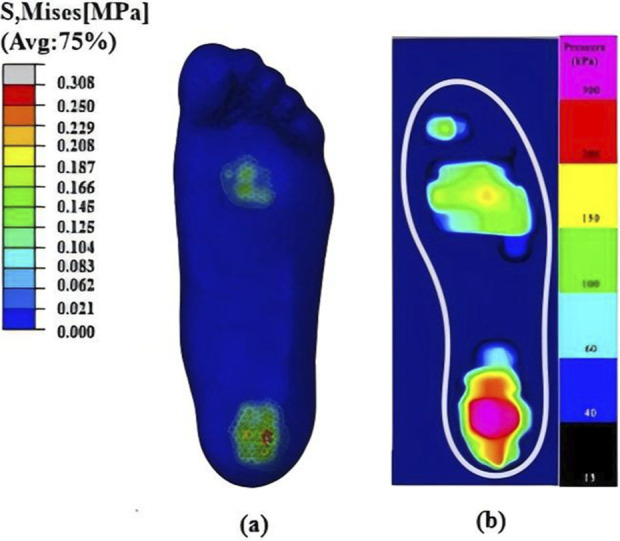
Comparison between FE predicted and experimentally measured pressure maps during static standing: **(a)** Numerical plantar pressures; **(b)** Experimental plantar pressures.

**FIGURE 9:**
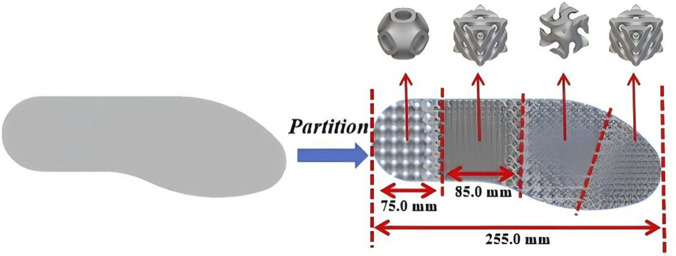
Schematic illustration of insole partitioning.

In order to determine the infilled partitions, the numerical simulation and experiment of static standing on the ground were performed. Numerical results were compared with experimental pressure maps, as shown in Figure 8. Similar overall patterns were observed, with plantar peak pressures at the heel and forefoot regions, and magnitudes within a comparable range. Regional characteristics of foot were indicated by combining FE analysis with experimental measurements. Peak pressures were concentrated at the heel and forefoot, whereas little concentration was detected at the toes and midfoot. These findings implied different functional requirements across regions. While maintaining overall stiffness and support, it is necessary to achieve effective pressure relief and cushioning in high-pressure regions, and to enhance structural stability and load-bearing capacity in low-pressure regions. Based on the results from FE simulation and experiment, a functional infilled design with partitions was proposed, as shown in Figure 9. The lowest stiffness Primitive lattice was infilled into the heel region to produce larger elastic deformation and increase contact area, thereby peak pressure was reduced and more impact was absorbed. The high stiffness Diamond lattice was infilled into the toe and midfoot regions to enhance load-bearing capacity and resistance to deformation, and thus to ensure geometric stability and sustained support. Through this partitioning strategy, a combination of pressure relief was achieved in the high-pressure regions with robust support in the low-pressure regions. The performance of the insole was increased under both static and dynamic loading conditions.

### 3.3 Evaluation of the performances of different TPMS infilled insoles

#### 3.3.1 Evaluation of the static performance

**FIGURE 10:**
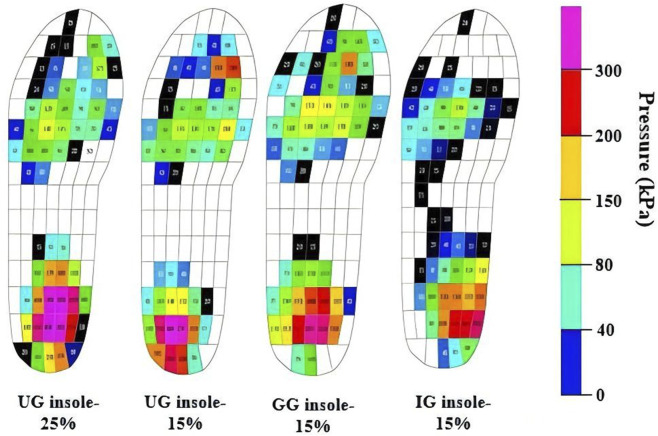
Experimental pressure maps during static standing with different orthotic insoles.

The static responsive performance for the PI insole-15, UG insole-15, and GG insole-15, and UG insole-25 were compared, as shown in Figure 10. Although average levels of plantar pressure for the UG insole-25 did not differ markedly from those of the partition-infilled insole, more exceptional peak values were observed. The UG insole-15 exhibited a maximum stress of 315.0 kPa, whereas the PI insole-15 exhibited 260.0 kPa. Thus, the PI design dispersed and mitigated local peak pressure more effectively while maintaining an overall load-bearing capacity. The PI insole-15 presented the most uniform distribution of pressure and the lowest peak values across the foot, with pressures in the forefoot and heel significantly lower than those of other insoles. Under the same static loadings, maximum pressure in the forefoot was reduced to below 200.0 kPa. Furthermore, for providing necessary support, PI insole-15 optimized the distribution of structural stiffness. The local region of PI insole-15 corresponding to midfoot region offered adequate rebound and stability, which helped maintain normal foot biomechanics. Additionally, the peak pressure of UG insole-25 was higher than that of UG insole-15, which demonstrated that the volume fraction was an essential factor to influence the peak pressure of insole. These results indicated superior ability of the insole to mitigate peak pressures under static standing, suggesting improved protection against plantar injury and better comfort during wearing.

**FIGURE 11:**
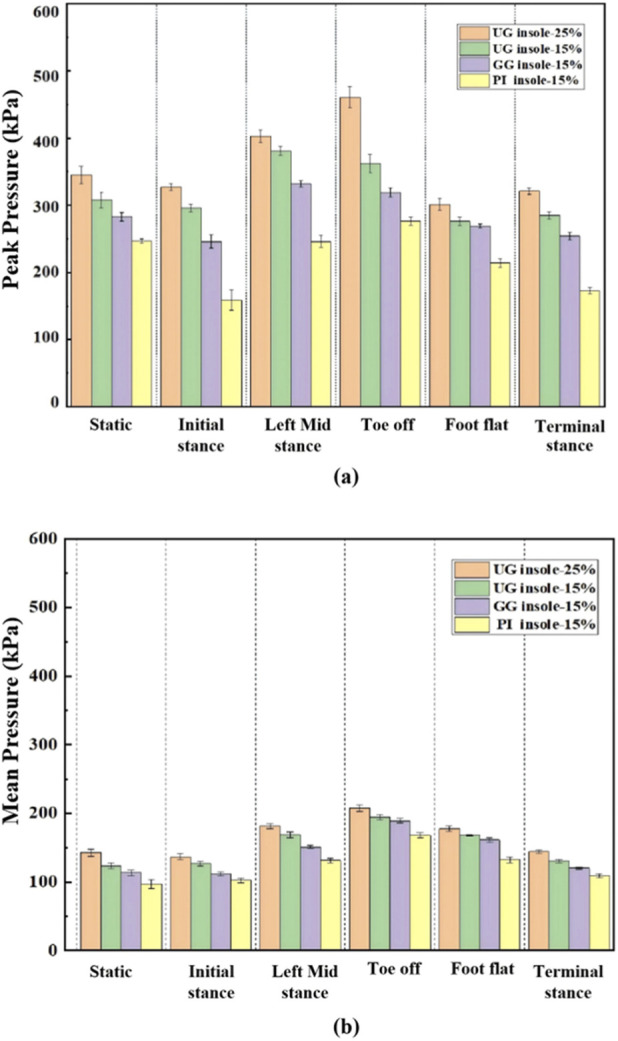
**(a)** Peak pressure when wearing different insoles; **(b)** Mean pressure when wearing different insoles.

The peak and mean pressures of PI insole-15, UG insole-15, GG insole-15, and UG insole-25 under static standing were compared in Figure 11. UG insole-25 consistently exhibited the highest peak pressures, and PI insole-15 consistently exhibited the lowest peak pressures, as shown in Figure 11a. Under static conditions, UG insole-25 exceeded UG insole-15, GG insole-15, and PI insole-15 by approximately 9.0%, 21.0%, and 36.0%, respectively. Trends of mean pressure matched those of peak pressure: UG insole-25 was the highest across all phases, PI insole-15 the lowest, with GG insole-15 generally slightly lower than UG insole-15. Under static conditions, PI insole-15 decreased mean pressure by 37.0% relative to UG insole-25, as shown in Figure 11b.

#### 3.3.2 Evaluation of the dynamic performance

**FIGURE 12:**
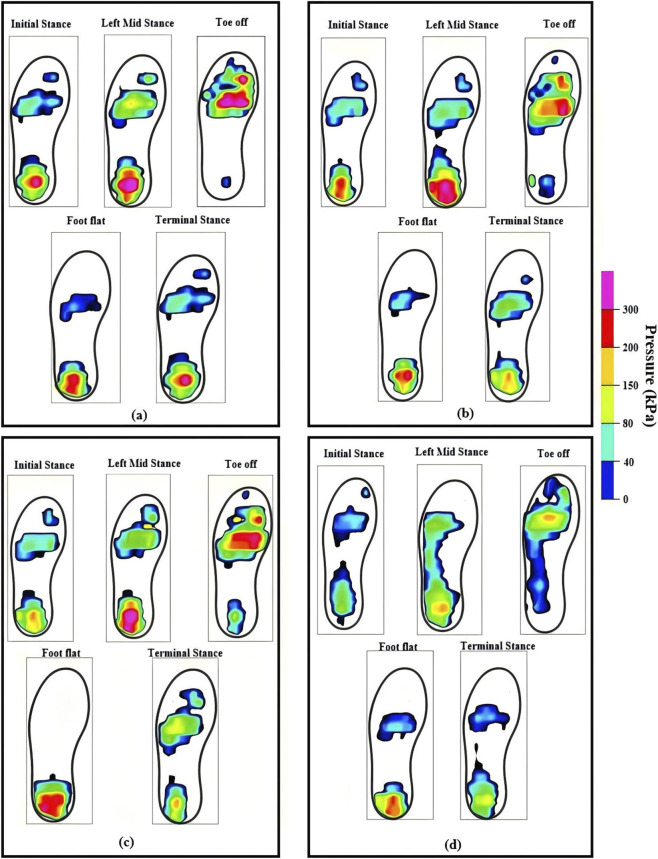
**(a)** Gait cloud diagram of UG insole-25; **(b)** Gait cloud diagram of UG insole-15; **(c)** Gait cloud diagram of GG insole-15; **(d)** Gait cloud diagram of PI insole-15.

The dynamic responsive performance for the PI insole-15, UG insole-15, and GG insole-15, and UG insole-25 were compared, as shown in Figure 12. The PI insole-15 displayed the most balanced pressure distributions and the lowest peak pressure across the gait cycle among these insoles. High-pressure regions of PI insole-15 reduced markedly, and its peaks were significantly lower than those of UG insole-15, and GG insole-15, and UG insole-25. In particular, regions above 200.0 kPa virtually disappeared at foot flat and toe-off, leaving mainly low-to-moderate pressures. The peak pressure of UG insole-15 mostly remained below 175.0 kPa, with only localized high-pressure spots at toe-off. The GG insole-15 exhibited peak value of 247.6 kPa (terminal stance), 239.0 kPa (foot flat), 338.0 kPa (mid stance), and 312.0 kPa (toe off). Although the UG insole-25 insole has low pressure in the midfoot region, it still had excessively pressure peaks in other regions at overall gait cycle. These results demonstrated that the novel PI insole exhibited relief capabilities of exceptional peak pressure during walking, meaning it more effectively protects the feet from injury and enhances comfort while worn.

The peak and mean pressures of PI insole-15, UG insole-15, GG insole-15, and UG insole-25 across each phase of walking were compared in Figure 11. At terminal stance, UG insole-25 exceeded UG insole-15, GG insole-15, and PI insole-15 by 10.0%, 23.0%, and 44.0%, respectively. The left mid-stance phase exhibited the highest peak pressures across all test samples, as shown in Figure 11a. Specifically, the UG insole-25, UG insole-15, GG insole-15, and PI insole-15 recorded the maximum pressure values during this phase. Furthermore, the UG insole-25 demonstrated a 49.0% increase in pressure compared to the PI insole-15. During the toe-off phase, the peak pressures followed a specific distribution across the UG insole-25, UG insole-15, GG insole-15, and PI insole-15 samples. Notably, the UG insole-25 exhibited a pressure value 41.0% higher than that of the PI insole-15. In the foot-flat phase, the UG insole-25, UG insole-15, GG insole-15, and PI insole-15 recorded the maximum pressures for this stage. Specifically, the UG insole-25 demonstrated an approximate 43.0% increase in pressure relative to the PI insole-15. The second terminal stance showed similar trends. On average, PI insole-15 reduced peaks by 42.0% relative to UG insole-25; UG insole-15 and GG insole-15 achieved 15.0%–25.0% and 25.0%–35.0% reductions, respectively. Similar relative reductions were observed in terms of the mean pressure, with PI insole-15 typically 30.0%–37.0% lower than UG insole-25% and 15.0%–25.0% and 10.0%–18.0% lower than UG insole-15 and GG insole-15, respectively, as shown in Figure 11b. PI insole-15 excelled at lowering global load, mitigating fatigue accumulation and risk of tissue damage over prolonged standing and walking. GG insole-15 balanced support and pressure reduction.

A total of five healthy male subjects were recruited (mean height 172 ± 2.3 cm, weight 65.7 ± 6.8 kg, foot length 24.6 ± 0.7 cm). Each subject performed three gait tests to enhance repeatability. Raw data were strictly filtered, and exceptional values due to unstable gait, posture deviations, or transient interference were removed. The average value of each phase was then computed. The peak and mean pressures of different regions are shown in Tables 2, 3. It should be noted that the foot FE model was derived from one of these five subjects. Multiple subjects, repeated tests, and rigorous processing mitigated the influence of individual variability and supported the generalizability and clinical potential of the proposed design.

**TABLE 2:** Peak value and standard deviation of plantar pressures in different static standings and gait of the foot for the five subjects (Unit: kPa).

Status	UG insole-25	UG insole-15	GG insole-15	PI insole-15
Static standings	247.23 ( ± 7.93)	237.67 ( ± 8.90)	204.67 ( ± 6.81)	186.81 ( ± 7.32)
Terminal stance	301.65 ( ± 15.33)	266.52 ( ± 9.11)	239.17 ( ± 7.21)	197.33 ( ± 9.89)
Left mid stance	399.38 ( ± 6.17)	378.42 ( ± 7.23)	356.61 ( ± 10.21)	309.89 ( ± 7.53)
Toe off	380.20 ( ± 8.71)	352.11 ( ± 9.01)	324.67 ( ± 5.89)	277.44 ( ± 10.20)
Foot flat	367.29 ( ± 5.97)	289.71 ( ± 10.23)	277.44 ( ± 7.00)	209.42 ( ± 8.02)
Terminal stance	291.45 ( ± 10.30)	246.42 ( ± 6.41)	238.04 ( ± 5.03)	167.57 ( ± 12.33)

**TABLE 3:** Mean value and standard deviation of plantar pressures in different static standings and gait of the foot for the five subjects (Unit: kPa).

Status	UG insole-25	UG insole-15	GG insole-15	PI insole-15
Static standing	137.44 ( ± 6.02)	119.22 ( ± 3.99)	104.54 ( ± 6.21)	96.70 ( ± 6.12)
Terminal stance	142.32 ( ± 12.13)	132.43 ( ± 6.85)	122.09 ( ± 5.41)	103.23 ( ± 4.29)
Left mid stance	169.21 ( ± 4.30)	160.20 ( ± 3.43)	136.61 ( ± 8.53)	104.29 ( ± 6.18)
Toe off	192.35 ( ± 6.83)	183.41 ( ± 5.32)	174.63 ( ± 6.11)	154.60 ( ± 7.24)
Foot flat	167.44 ( ± 8.23)	149.92 ( ± 4.89)	147.34 ( ± 8.02)	124.31 ( ± 6.23)
Terminal stance	141.45 ( ± 5.38)	134.47 ( ± 6.00)	138.14 ( ± 7.03)	103.27 ( ± 5.41)

## 4 Discussion

As a protective layer for the sole, insoles play a critical role in redistributing plantar loads and mitigating excessive local pressure, which represents a fundamental biomechanical strategy that may benefit populations at risk of foot complications. Conventional pressure-relief insoles typically rely on homogeneous materials or simplified stiffness gradients, which limits their ability to address region-specific biomechanical demands under both static and dynamic conditions. Although recent advances in additive manufacturing have enabled lattice structures with tunable mechanical properties, most existing designs employ single lattice types or continuous gradients, providing limited control over localized pressure redistribution. To address these limitations, this study proposed and systematically evaluated a partition-infilled functional insole based on TPMS lattices. By integrating plantar pressure distribution with the intrinsic mechanical characteristics of different TPMS architectures, the proposed design demonstrated superior overall pressure-relief performance compared with uniform-lattice configurations. Based on these findings, four main conclusions can be drawn, which are discussed in detail below.

First, the overall pressure-relief performance of partition-infilled TPMS insoles was significantly superior to that of uniform-lattice designs. The significant advantage of partition-infilled TPMS structures in reducing plantar pressure has been systematically verified. Relative to UG insole-25, introducing multiple TPMS lattices tailored to regional mechanical demands produced simultaneous and substantial reductions in both peak and mean pressures during static standing and dynamic gait. PI insole-15 achieved the best pressure relief among all designs. To clarify the mechanism underlying the superior pressure-relief performance observed above, quantitative comparisons demonstrate that regional partitioning plays a more critical role than simple wall-thickness reduction. Compared to UG insole-25, PI insole-15 significantly reduced mean pressure, with a 37.0% decrease in static mean pressure and a 30.0%–35.0% decrease in dynamic mean pressure. This indicates that its superior performance is not solely due to Gyroid wall thickness, as adjusting it alone only provided a limited 10.0%–15.0% reduction in mean pressure. The GG insole-15 achieved 18.0%–25.0% lower mean pressures than UG insole-25 during walking, consistent with the findings of Dayna Cracknell’s team (Cracknell et al., 2025), but its static pressure relief was noticeably weaker (10.0%–18.0%). Therefore, while gradients within a single lattice successfully improved dynamic response, they offered limited control over peak and mean pressures in static conditions. In contrast, partition filling with different TPMS types delivered the most comprehensive benefits.

Second, the superior pressure-relief performance of the PI insole can be attributed to the rational assignment of TPMS types according to regional biomechanical demands. In this study, the heel region was infilled with the lowest-stiffness Primitive lattice to enlarge deformation and contact area and thus reduce peak pressure. The forefoot region was infilled with Gyroid to disperse pressure while maintaining support, whereas the midfoot and toe regions were infilled with Diamond to ensure structural stability. Owing to the unique mathematical descriptions and parameterization of TPMS, smooth transitions between different lattices were enabled under macroscopic geometric continuity constraints, satisfying manufacturability while allowing region-specific tuning of mechanical response. These capabilities that are difficult to achieve with traditional honeycomb or triangular lattices.

Third, the superior pressure-relief performance of the proposed insole fundamentally stems from its strategic approach to optimize plantar load redistribution. It has been well documented that unloading peak-pressure regions shifts load toward the mid foot, creating a more uniform distribution and, despite higher mid foot loads, improving comfort. The essence of this study lies in transferring load from peak-pressure zones to lower-pressure areas by increasing contact area, thereby lowering both plantar peak and mean pressures.

Finally, the proposed partition strategy demonstrates a big potential for personalization despite current manufacturing constraints. A 0.6 mm wall thickness was adopted for the partition-filled TPMS due to printer resolution limits, which prevented further thinning. Prior studies have indicated that smaller wall thicknesses in high-pressure regions markedly enhance local deformability and cushioning, facilitating dispersion of concentrated pressure at the metatarsal heads and calcaneus. Unlike traditional insoles with homogeneous materials, our design leveraged the low-stiffness Primitive cells at the heel for impact absorption and the high-stiffness Diamond cells at the arch for structural integrity. The use of a Sigmoid-based blending function further ensured mechanical continuity between these heterogeneous zones, eliminating stress concentration at the interfaces. The experimental validation through both simulation and gait analysis proved that our partition infilling strategy effectively can mitigate the risk of deep tissue injury, providing a robust technical path for high-performance orthotic customization. Because DFU sites and pressure distributions vary greatly among patients, a single fixed infill strategy cannot satisfy all protection needs. The proposed framework provides adjustable structural parameters, allowing local wall thickness and infill density to be tuned according to each patient’s high-pressure regions, offering a structural foundation that may support personalized protection strategies in the future. However, it must be emphasized that these findings are currently limited to biomechanical evaluations on healthy subjects. The actual clinical efficacy for ulcer prevention, real-world tissue-damaging risks, and long-term protective performance require extensive future testing in diabetic patients.

While previous studies have explored multi-lattice designs, most state-of-the-art approaches primarily rely on adjusting the geometric parameters (e.g., cell size, wall thickness, or center offset) of a single lattice type, such as the Gyroid structure, to achieve regional stiffness variations (Feng et al., 2022; Jonnala et al., 2023). However, these single-topology graded designs may struggle to fully accommodate the complex, multi-axial stress states and extreme pressure distribution characteristics of diabetic foot biomechanics. Currently, research on integrating fundamentally different TPMS topologies within a single orthotic insole remains rare. Our proposed strategy advances beyond current approaches by utilizing a multi-topology design. The various TPMS structures, including Primitive, Gyroid, and Diamond, exhibit distinct initial elastic moduli and different mechanical advantages under the same geometric parameters. By employing a Sigmoid function to ensure seamless, stress-concentration-free transitions between these distinct topologies, our method offered a more rigorous and highly customizable structural solution for plantar pressure redistribution compared to conventional single-lattice graded designs.

Some limitations in the present work should be noted. First, the current study primarily focused on the partition strategy among different TPMS types, yet the optimization of wall thickness within each region was not fully explored due to manufacturing constraints. The fixed wall thickness, while necessary for printability, may not represent the optimal mechanical performance for each lattice type in their respective regions. Graded PI insole should be explored in the future works. Second, the quantitative validation using error metrics or statistical comparison between simulated and experimental plantar pressure distributions is lacking. While qualitative agreement was achieved, future work should focus on acquiring high-fidelity experimental data to rigorously evaluate the model’s predictive capabilities. Additionally, advanced mechanical characterizations, such as fatigue and dynamic testing, are lacking in the present study. The long-term cyclic performance of these TPMS structures and PI insoles beyond their basic static stiffness should be investigated in the future work. Third, the FE modelling is based on a single subject and incorporated structural simplifications, such as omitting ligaments, tendons, and cartilage, and treating soft tissue as a bulk material. While this approach captures general pressure redistribution trends, it decreases the physiological accuracy of localized stress analysis. Additionally, when applying these complex multi-lattice designs to real-world applications, manufacturing considerations are crucial and should be addressed. The current insoles were fabricated using FDM technology, which inherently introduces material anisotropy due to the layer-by-layer deposition process, resulting in weaker mechanical properties and lower inter-layer adhesion along the build direction (Z-axis). Furthermore, typical FDM printing defects, such as internal voids, under-extrusion, and the surface stair-stepping effect, can induce localized stress concentrations. These defects may compromise the fatigue resistance and long-term structural integrity of the intricate TPMS lattices under cyclic walking loads. Therefore, future research should also focus on optimizing printing parameters or exploring alternative high-resolution additive manufacturing techniques (e.g., Selective Laser Sintering, SLS) to mitigate these manufacturing-induced variations and ensure reliable real-world performance. Fourth, the current study employed a simplified foot model that treats soft tissue as a bulk material and omits internal structures such as ligaments and tendons. Because the simulations focus exclusively on static standing to evaluate macro-level foot-insole interactions, complex musculoskeletal loading conditions are not considered. While these modeling assumptions are sufficient in the present study, they will be addressed in future work through the development of high-fidelity anatomical foot models, the integration of musculoskeletal models to apply dynamic gait loads, and the execution of more comprehensive quantitative validations. Last but not the least, the experimental validation in this study was conducted on a small cohort of only five healthy male subjects. As plantar pressure distribution and soft tissue characteristics vary greatly between healthy individuals and diabetic patients, the current results primarily serve as an initial proof-of-concept. While the design allows for personalization, the current framework has not been validated across a wide range of patient-specific foot anatomies and pressure profiles. Future studies with larger sample sizes, specifically including diabetic populations, are essential to rigorously evaluate the clinical efficacy and real-world performance of these customized orthotic insoles. Furthermore, it should be noted that in the present study, the insole was design based on the plantar pressure distribution of the five subjects and the subject-specific lattice parameters for each subject are not considered. The partitioning strategy has not been adapted to different foot morphologies. These points and work need to be investigated in the future work.

## 5 Conclusion

In this study, a partition-infilled insole was designed based on TPMS lattices with a volume fraction of 15.0% and its performance was evaluated using both numerical analysis and experimental testing under static loading and gait analysis. Relative to traditional uniform and graded Gyroid structure infilled insoles, the partition-infilled insole further reduced peak pressures in high-pressure regions while a more uniform distribution of plantar pressure was achieved and stability was maintained. The pressure-relief advantage of TPMS lattices in high-pressure regions has been effectively exploited using partition strategy, offering a big potential for relieving the plantar pressure. Future work will integrate multi-scale FE modeling and clinical data to enable automated customization for different diabetic patients.

## Data Availability

The original contributions presented in the study are included in the article/supplementary material, further inquiries can be directed to the corresponding author.
